# Presentation pattern and management of effusive–constrictive pericarditis in Ibadan

**DOI:** 10.5830/CVJA-2011-066

**Published:** 2012-05

**Authors:** MA Salami, PO Adeoye, VO Adegboye, OA Adebo

**Affiliations:** Department of Surgery, Cardiovascular and Thoracic Surgery Division, University College Hospital and College of Medicine, University of Ibadan, Ibadan, Nigeria; Department of Surgery, Cardiovascular and Thoracic Surgery Division, University College Hospital and College of Medicine, University of Ibadan, Ibadan, Nigeria; Department of Surgery, Cardiovascular and Thoracic Surgery Division, University College Hospital and College of Medicine, University of Ibadan, Ibadan, Nigeria; College of Health Sciences, Bowen University, Iwo, Nigeria

**Keywords:** pericarditis, effusive, constrictive, Ibadan, African

## Abstract

**Background:**

Effusive–constrictive pericarditis is a syndrome in which constriction by the visceral pericardium occurs in the presence of a dense effusion in a free pericardial space. Treatment of this disease is problematic because pericardiocentesis does not relieve the impaired filling of the heart and surgical removal of the visceral pericardium is challenging. We sought to provide further information by addressing the evolution and clinico-pathological pattern, and optimal surgical management of this disease.

**Methods:**

We conducted a prospective review of a consecutive series of five patients managed in the cardiothoracic surgery unit of University College Hospital, Ibadan, in the previous year, along with a general overview of other cases managed over a seven-year period. This was followed by an extensive literature review with a special focus on Africa.

**Results:**

The diagnosis of effusive–constrictive pericarditis was established on the basis of clinical findings of features of pericardial disease with evidence of pericardial effusion, and echocardiographic finding of constrictive physiology with or without radiological evidence of pericardial calcification. A review of our surgical records over the previous seven years revealed a prevalence of 13% among patients with pericardial disease of any type (11/86), 22% of patients presenting with effusive pericardial disease (11/50) and 35% who had had pericardiectomy for constrictive pericarditis (11/31). All five cases in this series were confirmed by a clinical scenario of non-resolving cardiac impairment despite adequate open pericardial drainage. They all improved following pericardiectomy.

**Conclusion:**

Effusive–constrictive pericarditis as a subset of pericardial disease deserves closer study and individualisation of treatment. Evaluating patients suspected of having the disease affords clinicians the opportunity to integrate clinical features and non-invasive investigations with or without findings at pericardiostomy, to derive a management plan tailored to each patient. The limited number of patients in this series called for caution in generalisation. Hence our aim was to increase the sensitivity of others to issues raised and help spur on further collaborative studies to lay down guidelines with an African perspective.

## Abstract

Effusive–constrictive pericarditis is a clinical syndrome characterised by concurrent pericardial effusion and pericardial constriction where constrictive haemodynamics are persistent after the pericardial effusion is removed. The treatment of effusive–constrictive pericarditis is problematic because pericardiocentesis does not relieve the impaired filling of the heart, and surgical removal of the fibrinous exudate coating the visceral pericardium may not be possible.^1^ Pericardiectomy following development of a pericardial skin that is amenable to surgical stripping is usually the most successful treatment option. The objectives of this case series were to document the evolution and clinico-pathological pattern of this disease in Nigerians.

## Methods

We conducted a prospective review of a consecutive series of five patients managed in the cardiothoracic surgery unit of University College Hospital, Ibadan in the previous year, along with a general overview of other cases managed over a seven-year period. This was followed by an extensive literature review with a special focus on Africa. The diagnosis of effusive–constrictive pericarditis was established on the basis of clinical findings of features of pericardial disease with evidence of pericardial effusion, and echocardiographic finding of constrictive physiology with or without radiological evidence of pericardial calcification.

## Results

A review of our surgical records over the previous seven years revealed a prevalence of 13% among patients with pericardial disease of any type (11/86), 22% of patients presenting with effusive pericardial disease (11/50) and 35% who had pericardiectomy for constrictive pericarditis (11/31). The present subset was chosen for the prospective follow up due to the unusual consecutive presentation and a dearth of studies specifically on this subset of patients from Africa.

All five cases in this series were confirmed by a clinical scenario of non-resolving cardiac impairment despite adequate open pericardial drainage. All five patients were prospectively followed up. One patient, who we treated for effusive–contrictive pericarditis, is described in detail and four other cases are summarised in tabular form (Table 1).

**Table 1:** Summary Of Cases Of Effusive–Constrictive Pericarditis

*Patient*	*Age (years)*	*Gender*	*Comorbid conditions*	*HIV status*	*Initial procedure*	*Pericardial histology*	*Post-op NYHA*
1. SB Pre-op NYHA III	46	M	Superficial thigh wound from gunshot	Negative	Pericardial window and biopsy	Tuberculous pericarditis	II
2. DS Pre-op NYHA III	19	M	Haemoglobin AS	–	Pericardial window and biopsy	Non-specific calcific pericarditis	I
3. AO Pre-op NYHA IV	20	F	–	Negative	Pericardial window and biopsy	Non-specific chronic pericarditis	I
4. MN Pre-op NYHA IV	19	F	Endomyocardial fibrosis Tricuspid regurgitation	Negative	Pericardial window and biopsy	Pericardial fibrosis	I
5. OS Pre-op NYHA IV	20	M	Fournier’s gangrene Upper gastrointestinal bleeding	Negative	Pericardial window	Non-specific chronic pericarditis	I

## Case studies

A 20-year-old, HIV sero-negative lady presented to the cardiothoracic unit of the University College Hospital, Ibadan with a three-year history of easy fatigability, exertional dyspnoea and weight loss. There was a history of cough productive of whitish sputum. There was an associated history of orthopnoea, chest discomfort and bulging chest, but no history of leg swelling. The patient was wasted and afebrile with a respiratory rate of 32 breaths/min. Her blood pressure and pulse were, respectively, 105/80 mmHg and 102 per min. Her neck veins were distended and she had a bulging anterior chest and hepatomegaly.

**Fig. 1.**
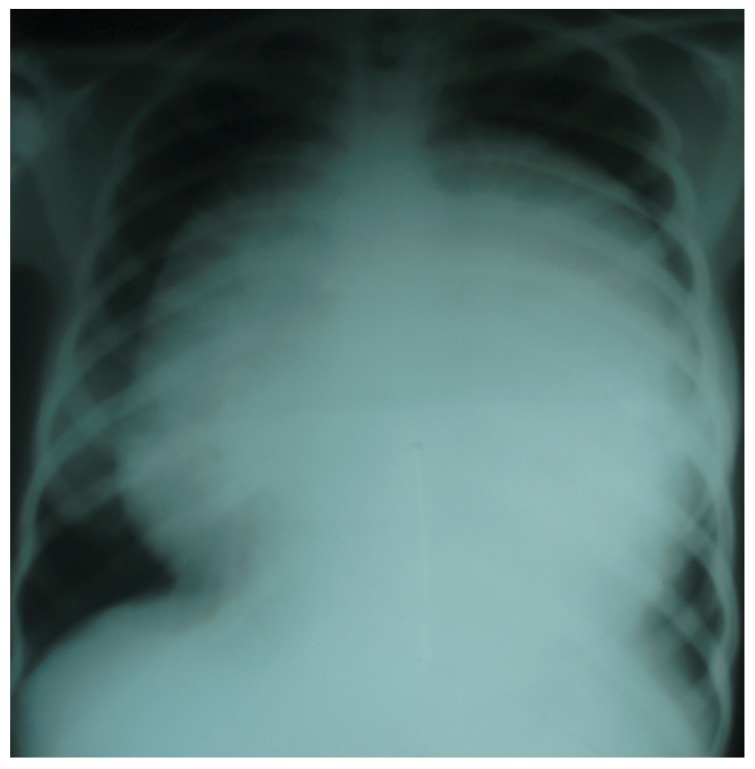
Radiograph showing massive globular heart shadow.

The patient’s packed cell volume was 40%. Her blood chemistry findings were normal. The chest radiograph showed a globular heart shadow (Fig. 1). The ECG revealed low-voltage waves. An echocardiogram revealed a large pericardial effusion with echo speckles within it and a thickened pericardium. There was septal bounce and a dilated inferior vena cava with blunted respiratory fluctuations in diameter. A diagnostic pericardiocentesis yielded serosanguinous fluid.

**Fig. 2.**
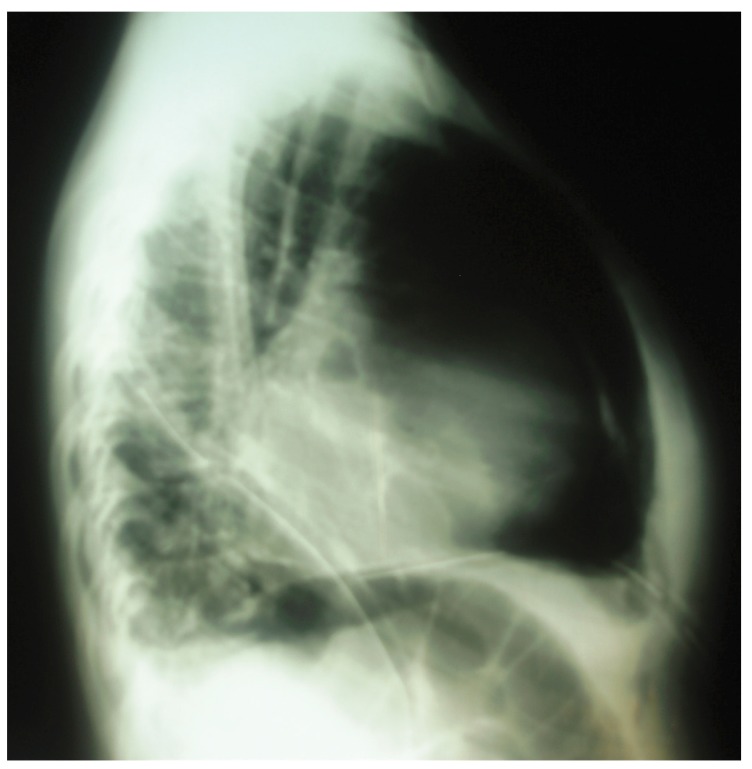
Radiograph showing evidence of pericardial calcification.

The patient underwent a subxiphoid tube pericardiostomy with pericardial biopsy. A postoperative chest radiograph showed evidence of pericardial calcification (Fig. 2). She was scheduled for an elective pericardiectomy, which was declined. The pericardiostomy tube was removed one week post operation. A subsequent radiograph revealed evidence of re-accumulation of pericardial fluid. The patient and her relatives still declined surgery and asked for a discharge.

She represented about 48 hours later with evidence of massive pericardial effusion and cardiac tamponade. She then had an emergency pericardiocentesis under echocardiographic guidance, during which 1 940 ml of haemorrhagic effusion was aspirated and another 2 250 ml four days later. She improved following this and then had a pericardiectomy.

**Fig. 3.**
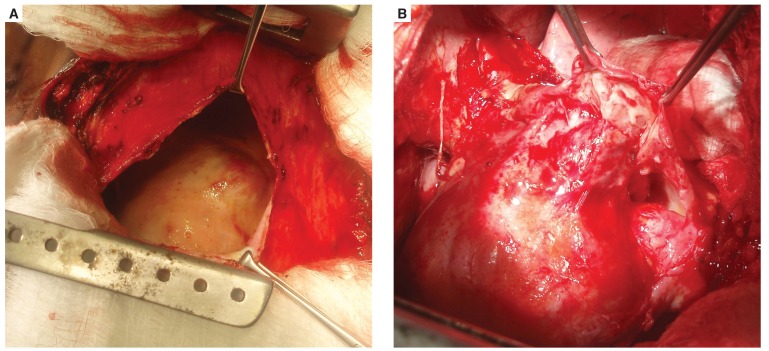
(A) Thickened pericardium and a large pericardial space. (B) Final phase of visceral pericardial stripping.

Findings at surgery included a thickened parietal and visceral pericardium, about 1.5 l of serosanguinous fluid in the pericardial space, and an area of calcification particularly over the right atrium (Fig. 3A, B). Both the parietal and visceral pericardium were stripped. The patient had an uneventful postoperative recovery period and was discharged home 10 days after surgery. She has been seen twice since discharge, the last visit eight months post operation, with remarkable recovery, and NYHA class I status.

SM had a pre-operative (pericardial window) echo, which showed effusion with constrictive physiology. He had modest postoperative improvement and was discharged but he represented three months later with worsening of pre-operative symptoms. He then had a pericardiectomy, following which he improved progressively.

**Fig. 4.**
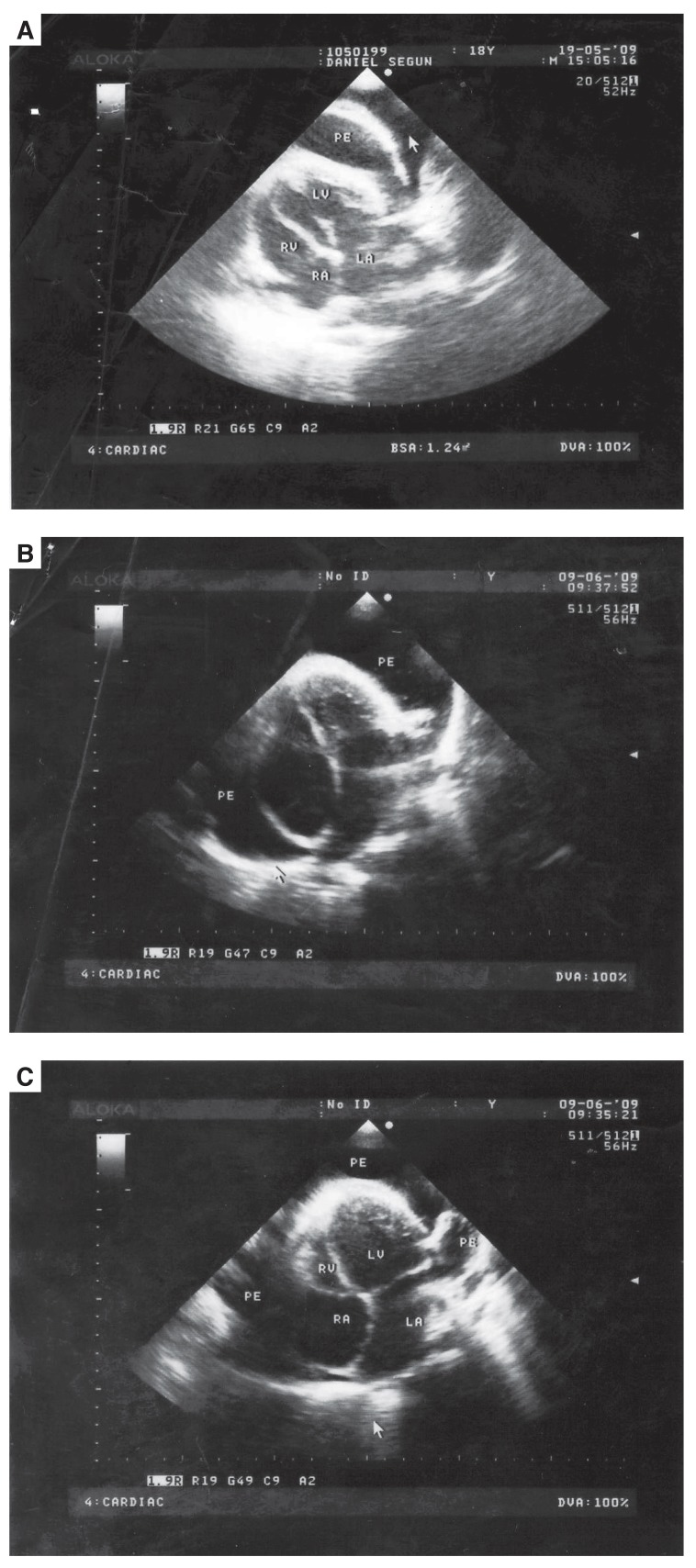
Echocardiography showing moderate pericardial effusion (PE). RV = right ventricle; LV = left ventricle; RA = right atrium; LA = left atrium.

Following tube pericardiostomy, DS had very transient improvement in his symptoms. Repeat lateral chest X-ray showed evidence of pericardial calcification while echocardiography showed moderate pericardial effusion and diastolic dysfunction (Fig. 4). He made a rapid recovery following pericardiectomy.

MN had minimal improvement following tube pericardiostomy, remaining dyspnoeic at rest. Postoperative chest radiography and echocardiography showed pericardial calcification. In addition, there was a markedly enlarged right atrium, grade III–IV tricuspid regurgitation and a small right ventricle with endocardial thickening, suggestive of endomyocardial fibrosis. We elected to go ahead with a pericardiectomy on account of the pericardial thickening with calcification. She improved following pericardiectomy, with NYHA class I status.

OS had pericardiostomy with slight improvement and was discharged home on anti-tuberculous therapy. He had a pericardiectomy three months later, during which he had an intra-operative complication of right ventricular wall injury, which was promptly repaired. He had an uneventful postoperative recovery until the 12th and 19th days postoperatively, when he developed Fournier’s gangrene and upper gastrointestinal bleeding, respectively. These were successfully managed and he was discharged home on the 36th day postoperatively.

## Discussion

Effusive–constrictive pericarditis is said to be an uncommon pericardial syndrome.^2^ In a prospective study of 1 184 patients with pericarditis, Sagrista-Sauleda *et al*. reported a prevalence of only 1.3% among patients with pericardial disease of any type (15/1 184) and 6.9% among patients with clinical tamponade (15/218).^3^ However, a recent observational study by Mayosi *et al*. reported 28 (15.1%) of 185 patients with tuberculous pericarditis as belonging to that subset.^4^ This is quite similar to the prevalence of 13% among patients with pericardial disease of any type in our seven-year review (11/86). We are not aware of any specific series from Africa.

Patients with effusive–constrictive pericarditis present with symptoms due to limitation of diastolic filling. These findings are secondary not only to the pericardial effusion but also the pericardial constriction. Symptoms and physical findings vary, while a moderate-to-large pericardial effusion may occur. Management of effusive–constrictive pericarditis is therefore fraught with challenges.

The diagnosis is usually made by echocardiography, which should demonstrate diastolic dysfunction. The diagnosis can easily be missed by an unwary clinician because of the usual superimposed features of accompanying pericardial effusion or tamponade. This may have accounted for the premature discharge and re-admission of one of our patients (SB).

Pericardial effusion is seen as an echo-free space around the heart on echocardiography (Fig. 4). The presence of a large pericardial effusion with frond-like projections and a thick ‘porridge-like’ exudate is suggestive of an exudate but not specific for a tuberculous aetiology.^1^ Patients with acute haemorrhagic effusions may have pericardial thrombus appearing as an echo-dense mass.^5^

Small pericardial effusions are only seen posteriorly, while those large enough to produce cardiac tamponade are usually circumferential. In large pericardial effusions, the heart may move freely within the pericardial cavity (‘swinging heart’). In the parasternal long-axis view, pericardial fluid reflects at the posterior atrio-ventricular groove, while pleural fluid continues under the left atrium, posterior to the descending aorta. Rarely, tumour masses are found within or adjacent to the pericardium and may masquerade as tamponade.^6^

Diagnostic criteria for cardiac tamponade include diastolic collapse of the right atrial and ventricular anterior free wall, and left atrial and very rarely left ventricular collapse. Right atrial collapse is more sensitive for tamponade, but right ventricular collapse lasting more than one-third of diastole is a more specific finding for cardiac tamponade. Doppler findings include distension of the inferior vena cava that does not diminish with inspiration, which is a manifestation of the elevated venous pressure in tamponade.^6^ In addition, there can be marked reciprocal respiratory variation in mitral and tricuspid flow velocities. Tricuspid flow increases and mitral flow decreases during inspiration (the reverse in expiration).

A challenging differential diagnosis is endomyocardial fibrosis, a common form of restrictive cardiomyopathy (RCM) in Africa.^7^ Because constrictive pericarditis can be corrected surgically, it is important to distinguish chronic constrictive pericarditis from restrictive cardiomyopathy, which has a similar physiological abnormality, i.e. restriction of ventricular filling. Helpful in the differentiation of these two conditions are right ventricular trans-venous endomyocardial biopsy (by revealing myocardial infiltration or fibrosis in RCM) and echocardiography, CT scan or cardiac magnetic resonance imaging (by demonstrating a thickened pericardium in constrictive pericarditis but not in RCM).^8^ Our fourth patient (MN) actually presented this challenge but a convincing thickening of the pericardium at echocardiography was enough to help us clarify the diagnosis.

Another important problem is the lack of placebo-controlled trials from which appropriate therapy may be selected, and of guidelines that assist in important clinical decisions. As a result, the practitioner must rely heavily on clinical judgment.^9^ The absence of guidelines specific to this subset of pericardial disease may be due to its relative rarity in the Western world. The recent European Society of Cardiology guidelines on management of pericardial diseases was also silent on the subset of patients with effusive constrictive pericarditis, presumably due to a paucity of data on the subject.^6^ Other reasons could be difficulty in reaching a diagnosis and varied aetiopathogenesis, necessitating different evolution patterns.

While there is an abundance of diagnostic armamentarium in the West, practitioners in sub-Saharan Africa largely have to cope with severe limitations in diagnostic facilities. An exception to this may be South Africa, where a recent report highlighted the value of contrast-enhanced magnetic resonance imaging (MRI) in delineating epicardial and pericardial inflammation in effusive–constrictive pericarditis.^10^ Cost is still an issue even if MRI becomes widely available. Clinical acumen and reasoning therefore still form the bedrock of clinical practice in most centres.

The cases managed in this series illustrate this point. In only two of the five cases was there a hint of constrictive physiology at the initial echocardiography, even though it is known there is a phase of transient sub-acute constriction, which may improve after pericardial drainage and medical treatment, especially with anti-tuberculous therapy in those arising secondary to tuberculosis. The only strong evidence of a high likelihood of need for pericardiectomy was the duration of the history in the first three patients. They all had a history longer than two years, suggestive of a chronic process.

Reaching an aetiological diagnosis is a real challenge globally but more problematic in our local practices. The results of pericardial fluid culture are frequently falsely negative and pericardial biopsy has a higher yield of diagnostic specimens.^11^-^13^ One therefore has to rely on pericardial tissue biopsy microbiology and histology. None of our patients had positive evidence from pericardial fluid microbiology or cytology. The histology of their pericardia is shown in Table 1. Three of the patients were therefore treated empirically with anti-tuberculous therapy.

The difficulty in establishing a bacteriological or histological diagnosis is foremost among unresolved issues in patients with pericarditis.^14^ A definite or proven diagnosis is based on demonstration of tubercle bacilli in the pericardial fluid or on histological section of the pericardium. A probable or presumed diagnosis is based on proof of tuberculosis elsewhere in a patient with otherwise unexplained pericarditis, a lymphocytic pericardial exudate with elevated biomarkers of tuberculous infection, and/or appropriate response to a trial of anti-tuberculosis chemotherapy.

The diagnostic difficulty is best demonstrated by a recent series of patients with tuberculous pericarditis where most patients were treated on clinical grounds, with microbiological evidence of tuberculosis obtained in only 13 (7.0%) patients.^4^ Hence, the focus currently is on indirect tests for tuberculous infection, including ADA levels and more importantly, lysozyme or IFN-γ assay, which appears to hold promise for reaching diagnosis of cases arising secondary to tuberculosis.^14^-^18^ Technical and financial constraints may, however, limit the diagnostic utility of IFN-γ in many developing countries.^1^ These tests are currently not available in our centre.

The importance of recognising the haemodynamic syndrome of tamponade and constriction characteristic of effusive–constrictive pericarditis lies in an acknowledgment of the contribution of the visceral layer of the pericardium to the pathogenesis of constriction and of the need to remove it surgically. However, not only is it sometimes surgically challenging to do an epicardectomy in some patients due to a flimsy, fibrinous visceral pericardium with attendant risk of haemorrhage; some patients may recover with medical treatment alone – so-called transient effusive–constrictive pericarditis.^3^,^19^ Three of the patients in this series actually had intra-operative haemorrhage from atrial or ventricular injury during the epicardectomy part of the procedure.

Visceral pericardiectomy is therefore a much more difficult and hazardous procedure than parietal pericardiectomy, but it is necessary for a good clinical result in cases of effusive–constrictive pericarditis. The clinical decision as to which patients need to be observed on medical treatment depends on presumed or confirmed aetiology, timing of presentation, and response to medical therapy.

## Decision based on aetiology

Causes of effusive–constrictive pericarditis are varied and usually practice-dependent. Tuberculosis is said to be responsible for approximately 70% of cases of large pericardial effusion and most cases of constrictive pericarditis in developing countries. However, in industrialised countries, tuberculosis accounts for only 4% of cases of pericardial effusion and an even smaller proportion of instances of constrictive pericarditis.^14^ Series from Europe and North America report a predominance of idiopathic cases, followed by cases that occur after radiotherapy or cardiac surgery, or as a result of neoplasia or tuberculosis.^3^,^11^,^20^

The aetiological spectrum indeed reflects the general aetiological spectrum of pericardial diseases in each area and can be influenced by the changing aetiological spectrum of pericarditis in general and constrictive pericarditis in particular.^3^,^21^,^22^ The varying aetiological spectrum impacts on the need for and timing of pericardiectomy.^17^

In the Sagrista-Sauleda series, pericardiectomy was not performed in eight of 15 patients; in five of them owing to a poor general prognosis (four patients with neoplastic pericarditis) or a high surgical risk (one patient with radiation pericarditis), and in three patients (all with idiopathic pericarditis) because of progressive improvement and eventually resolution of the illness after pericardiocentesis. Wide anterior pericardiectomy was performed in seven patients between 13 days and four months after pericardiocentesis owing to the persistence of severe right heart failure. The diagnoses in these seven patients were idiopathic pericarditis in four, radiation pericarditis in one, tuberculous pericarditis in one, and postsurgical pericarditis in one.

The patients in our limited series, as in others cases due to tuberculosis, usually had attendant pericardial calcification with no room for improvement without pericardectomy. This partly explains the need for pericardectomy in these patients.

## Decision based on timing of presentation and response to medication

Related to aetiology is the timing of presentation. Transient sub-acute effusive–constrictive pericarditis is known to resolve after pericardiocentesis without the need for pericardiectomy.^3^,^23^,^24^ In fact in two of three patients with idiopathic pericarditis who had resolution of their symptoms following pericardiocentesis in the Sagrista-Sauleda series, the onset of their illness was stated to be very recent. The monitoring of intra-cardiac and intra-pericardial pressures as part of a pericardiocentesis procedure has been suggested in patients who present with a sub-acute course of pericardial tamponade, particularly those in whom the condition is idiopathic or is related to infection, neoplasm or rheumatological disease.^2^

The duration of pericardial disease in three of our patients was more than two years, suggesting chronicity and need for pericardectomy. Although the duration in the fourth and fifth patients was relatively short, non-resolution of their symptoms and presence of pericardial calcification in the fourth patient appeared to be a predictor of need for pericardial stripping.

## Management

**Fig. 5.**
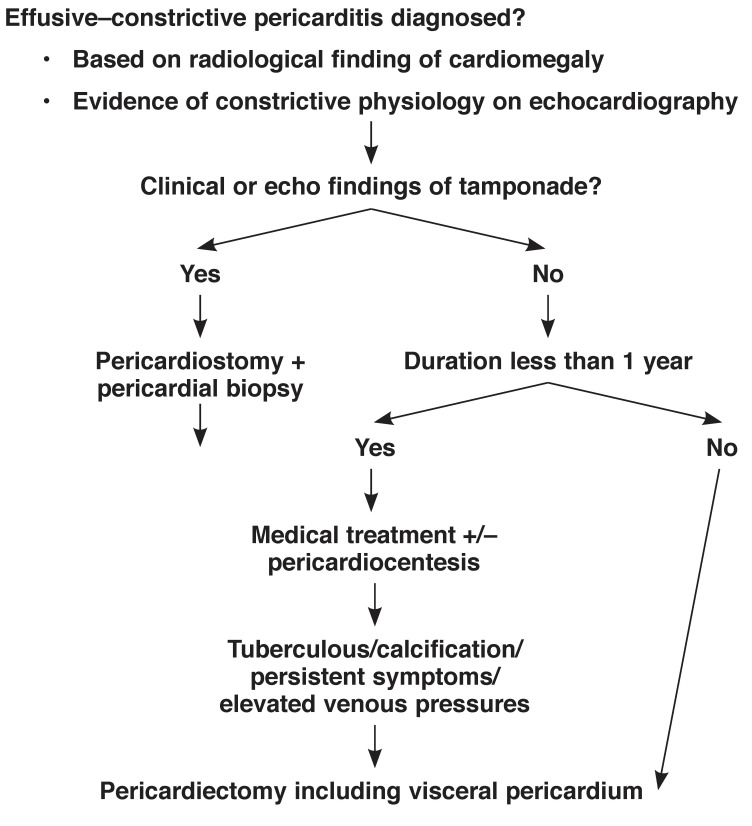
Potential algorithm for the management of effusive–constrictive pericarditis.

One can propose a management algorithm from the above discussion (Fig. 5). We would suggest pericardiocentesis followed by pericardiostomy and pericardial biopsy for bacteriology and histology as a first step in patients with tamponade or imminent tamponade. Duration of illness should be the next guide in those without tamponade, with those patients with duration more than one year offered pericardiostomy and biopsy. Other patients could be tried on medical treatment for six to eight weeks and operated on when there is persistent evidence of constriction. Presence of pericardial thickening with calcification following pericardiocentesis is an absolute indicator of need for a pericardiectomy. This can be further confirmed on a cardiac CT scan.

We believe this management algorithm is preliminary at best and is subject to improvement with more collaborative research. The current on-going multicentre study on the role of steroids in the prevention of constrictive pericarditis, involving centres in South Africa, Nigeria and other African countries, is one such study.^4^ Other studies could focus on influence of aetiology and duration of pericardial disease on the need for pericardiectomy in other areas.

## Conclusion

Effusive–constrictive pericarditis as a subset of pericardial disease deserves closer study and individualisation of treatment. Evaluating patients suspected of having the disease affords clinicians the opportunity to integrate clinical features and non-invasive investigations with or without findings at pericardiostomy to expeditiously arrive at a patient-specific management plan. The limited number of patients in this series is a limitation, which calls for caution in generalisation. Hence our aim was to increase the sensitivity of others to issues raised and help spur on further collaborative studies to lay down guidelines with an African perspective.

## References

[1] Mayosi BM, Burgess LJ, Doubell AF (2005). Tuberculous pericarditis.. Circulation.

[2] Hancock EW (2004). A clearer view of effusive-constrictive pericarditis.. Circulation.

[3] Sagristà-Sauleda J, Angel J, Sánchez A, Permanyer-Miralda G, Soler-Soler J (2004). Effusive-constrictive pericarditis.. N Engl J Med.

[4] Mayosi BM, Wiysonge CS, Ntsekhe M, Volmink JA, Gumedze F, Maartens G (2006). Clinical characteristics and initial management of patients with tuberculous pericarditis in the HIV era: the Investigation of the Management of Pericarditis in Africa (IMPI Africa) registry.. BMC Infect Dis.

[5] Knopf WD, Talley JD, Murphy DA (1987). An echo-dense mass in the pericardial space as a sign of left ventricular free wall rupture during acute myocardial infarction.. Am J Cardiol.

[6] (2004). Guidelines on the Diagnosis and Management of Pericardial Diseases Executive Summary.. Eur Heart J.

[7] Bukhman G, Ziegler J, Parry E (2008). Endomyocardial fibrosis: still a mystery after 60 years.. PLoS Negl Trop.

[8] Wynne J, Braunwald E, Fauci AS, Braunwald E, Kasper DL, Hauser SL, Longo DL, Jameson JL, Loscalzo J (2008). Cardiomyopathy and myocarditis.. Harrison’s Principles of Internal Medicine.

[9] Hoit BD (2002). Management of effusive and constrictive pericardial heart disease.. Circulation.

[10] Russell JBW, Syed FF, Ntsekhe M, Mayosi BM, Moosa S, Tshifularo M, Smedema JP (2008). Tuberculous effusive-constrictive pericarditis.. Cardiovasc J Afr.

[11] Fowler NO (1991). Tuberculous pericarditis.. J Am Med Assoc.

[12] Gooi HC, Smith JM (1978). Tuberculous pericarditis in Birmingham.. Thorax.

[13] Trautner BW, Darouiche RO (2001). Tuberculous pericarditis: Optimal diagnosis and management.. Clin Infect Dis..

[14] Mayosi BM, Syed FF (2007). A modern approach to tuberculous pericarditis.. Prog Cardiovasc Dis.

[15] Aggeli C, Pitsavos C, Brili S, Hasapis D, Frogoudaki A, Stefanadis C (2000). Relevance of adenosine deaminase and lysozyme measurements in the diagnosis of tuberculous pericarditis.. Cardiology.

[16] Burgess LJ, Reuter H, Carstens ME, Taljaard JJ, Doubell AF (2002). The use of adenosine deaminase and interferon-gamma as diagnostic tools for tuberculous pericarditis.. Chest.

[17] Reuter H, Burgess L, van Vuuren W, Doubell A (2006). Diagnosing tuberculous pericarditis.. Q J Med.

[18] Tuon FF, Litvoc MN, Lopes MI (2006). Adenosine deaminase and tuberculous pericarditis-A systematic review with meta-analysis.. Acta Tropica.

[19] Oh JK, Hatle LK, Mulvagh SL, Tajik AJ (1993). Transient constrictive pericarditis: diagnosis by two-dimensional Doppler echocardiography.. Mayo Clin Proc.

[20] Hancock EW (1971). Subacute effusive-constrictive pericarditis.. Circulation.

[21] Cameron J, Oesterle SN, Baldwin JC, Hancock EW (1987). The etiologic spectrum of constrictive pericarditis.. Am Heart J.

[22] Ling LH, Oh JK, Schaff HV, Danielson GK, Mahoney DW, Seward JB (1999). Constrictive pericarditis in the modern era: evolving clinical spectrum and impact on outcome after pericardiectomy.. Circulation.

[23] Woods T, Vidarsson B, Mosher D, Stein JH (1999). Transient effusive-constrictive pericarditis due to chemotherapy.. Clin Cardiol.

[24] Tanaka K, Kawauchi M, Murota Y (2002). Reversible subacute effusive-constrictive pericarditis after correction of double-chambered right ventricle: a case report.. J Cardiol.

